# Systematic Review and Meta-Analysis of Incidence and Prevalence of Endometriosis

**DOI:** 10.3390/healthcare9010029

**Published:** 2020-12-30

**Authors:** Antonio Sarria-Santamera, Binur Orazumbekova, Milan Terzic, Alpamys Issanov, Chen Chaowen, Angel Asúnsolo-del-Barco

**Affiliations:** 1 Department of Medicine, Nazarbayev University School of Medicine, Zhanibek-Kerey Khans Street, 5/1, Nur-Sultan 010000, Kazakhstan; binur.orazumbekova@nu.edu.kz (B.O.); milan.terzic@nu.edu.kz (M.T.); alpamys.issanov@nu.edu.kz (A.I.); 2 Spanish Network of Health Services Research and Chronic Diseases, REDISSEC, 28029 Madrid, Spain; 3 Instituto Mixto de Investigación Escuela Nacional de Sanidad-Universidad Nacional de Educación a Distancia, IMIENS-UNED, 28029 Madrid, Spain; 4 Clinical Academic Department of Women’s Health, National Research Center of Mother and Child Health, University Medical Center, Turan Ave. 32, Nur-Sultan 010000, Kazakhstan; 5 Department of Obstetrics, Gynecology and Reproductive Sciences, University of Pittsburgh School of Medicine, Pittsburgh, PA 15213, USA; 6 Department of Surgery, Medical and Social Sciences, Faculty of Medicine, University of Alcalá, Alcalá de 288871 Henares, Spain; ccwxibanya@163.com (C.C.); angel.asunsolo@uah.es (A.A.-d.-B.); 7 Department of Epidemiology and Biostatistics, City University of New York (CUNY) Graduate School of Public Health and Health Policy, New York, NY 10028, USA; 8 Ramón y Cajal Institute of Healthcare Research (IRYCIS), 28034 Madrid, Spain

**Keywords:** endometriosis, epidemiology, statistics, numerical data

## Abstract

There is still much controversy regarding the epidemiology of endometriosis. The objective of this work is to conduct a systematic review, and if possible, proceed with a meta-analysis of studies that have analyzed the incidence and prevalence of this condition among women in the general population. The inclusion criteria were papers published after 1997 that had reported data of the incidence or prevalence of endometriosis. The PubMed search engine was used to identify papers meeting the inclusion criteria from 1997 to 2019, with an additional manual search for the identification of potentially eligible studies. The search was limited to papers published in English. The risk of bias was assessed according to the Joanna Briggs Institute Critical Appraisal Checklist. As a result, 27 papers, which included a total of 28,660,652 women, were classified according to the type of design and sources of information in five subgroups. Pooled estimates of prevalence for studies with self-reported data were 0.05 (95% CI: 0.03; 0.06), 0.01 for population-based integrated information systems (95% CI: 0.01; 0.02), and 0.04 (95% CI 0.04; 0.05) in studies using other designs. The pooled incidence rate of endometriosis was: 1.36 per 1000 person-years (PY) (95% CI: 1.09; 1.63) for studies based on hospital discharges, 3.53 per 1000 PY (95% CI: 2.06; 4.99) for cohort studies, and 1.89 per 1000 PY (95% CI: 1.42; 2.37) for population-based integrated information systems. Meta-analysis indicated high heterogeneity based on I-squared statistics. This significant variability may not only be due to methodological issues and the specific limitations of the different designs and data analyzed, including case definitions and subject selection strategies, but also to the inherent heterogeneity of endometriosis. Epidemiological studies with appropriate study designs remain necessary to provide a valid estimation of the population burden of endometriosis.

## 1. Introduction

Endometriosis is a complex clinical condition [1] characterized by the growth of endometrial-like tissue, glands, and stroma outside of the uterine cavity. Endometriosis is the leading cause of morbidity among premenopausal women, often painful and chronic, and has a negative impact on a patient’s physical and emotional well-being, quality of life, and productivity, placing a significant economic and social burden on patients, their families, and society as a whole [2].

Despite extensive research commencing 160 years ago [3], there remain much controversy and dilemmas regarding this complex and enigmatic disorder, related to its etiopathogenesis, diagnosis, overall clinical management, prognosis, and the epidemiology of this condition. Since the early work of Eskenazi [4], there is an overall assumption that the prevalence of endometriosis is around 10%. That study reviewed data on the epidemiology of the endometriosis population based on three publications that reported the prevalence of endometriosis in the general population: (1) Vessey revealed the prevalence of endometriosis in a group of women attending family planning clinics to be 1.8% [5]; Houston, based on data from residents in Rochester, obtained incidence ranging between 108.8 and 246.9 per 100,000 women-year and estimated an annual prevalence between 2.5 and 8.2% [6]; and Kjerulff, analyzing data from the US Health Interview Survey, obtained a self-reported prevalence of 6.9 per 1000 [7].

After that publication, multiple authors have pursued to clarify the epidemiology of endometriosis. However, those publications used significantly different data sources and designs and were based on different definitions of an endometriosis case. The consequence is that regardless of the significant body of publications available, the controversy regarding the burden of this disease remains unresolved. The objective of this paper is to provide a systematic assessment of the existing evidence regarding the incidence and prevalence of endometriosis and, if possible, quantify the available evidence through a meta-analysis and provide a critical assessment of the advantages and limitations of the different methodological approaches and case definitions.

## 2. Materials and Methods

### 2.1. Search Strategy and Inclusion Criteria

The inclusion criteria for this systematic review were papers published after the Eskenazi paper through 31/8/2019 that had reported data of the incidence or prevalence of endometriosis, referring to the general population, published in English [2]. Papers that calculated incidence or prevalence in groups of women defined by attending specific clinical settings or in groups of women with certain health problems, such as those seeking treatment for infertility or other conditions that may be associated with endometriosis, such as gynecological cancers, were excluded. PubMed search engines were used to identify papers meeting the inclusion criteria.

### 2.2. Methods of the Review

The selection process was performed by 2 independent researchers (A.S.-S. and A.S.-d.-B.). Search results were screened based on the title and abstract. In the next stage, full-text articles of potentially suitable articles were obtained and assessed for the eligibility criteria: (1) the study population consisted of women with endometriosis; (2) women represented the general population. Studies that included exclusively selected groups of women seeking care for certain specific problems (that may be related to endometriosis) were excluded.

### 2.3. Data Extraction

The information was retrieved by two authors from selected articles to populate the a priori prepared tables, with the following columns: years of follow up, setting, population of reference, age, source of information, method of diagnosis, and incidence or prevalence.

The PRISMA chart was used for the illustration of search results. Additionally, the reference lists of all eligible papers for systematic review were manually checked for the possibility of potentially suitable papers.

### 2.4. Assessment for Risk of Bias

The quality of the different papers estimating the prevalence of the endometriosis included in this systematic review was evaluated following the checklist proposed by the Joanna Briggs Institute Critical Appraisal Checklist [8]. Papers with a score up to 49% reporting “yes” indicated a “high” risk of bias, 50–69% indicated a moderate risk of bias, and a score of 70% or higher reporting “yes” belonged to a low risk of bias.

### 2.5. Statistical Analysis

With the data obtained, to take into account possible between-study heterogeneity, random-effects meta-analysis models were calculated to obtain pooled estimates of the incidence and prevalence of endometriosis, assuming that the estimated identified in the studies included in the review were only a random sample of all possible effects. The incidence rate of endometriosis was reported per 1000 person-years (PE) for comparability within studies. The heterogeneity of included studies was assessed by I-squared statistics with *p* < 0.05 indicating significant results, and funnel plots were used to assess for the presence of publication bias. All statistical analyses were conducted using STATA 15.0 statistical software (StataCorp, College Station, TX, USA) [9].

## 3. Results

**Figure 1:**
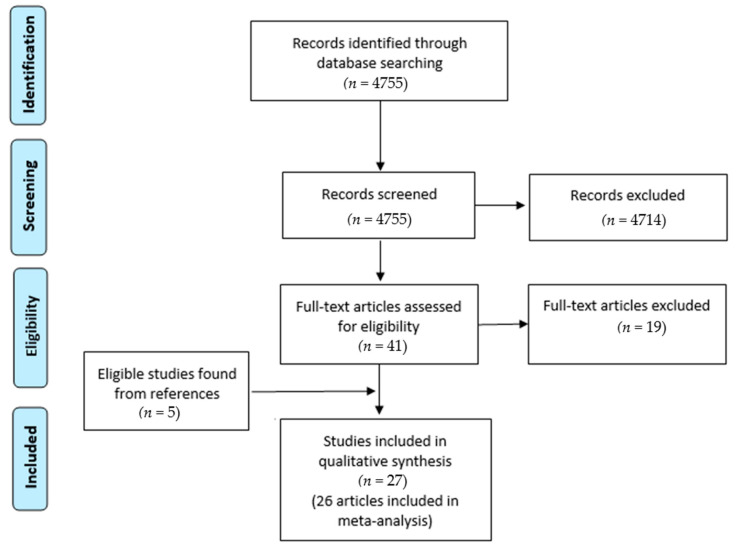
PRISMA flowchart.

Figure 1 shows the flowchart search from 1997 through to 31 August 2019. A total of 4755 articles were obtained. Based on the titles and abstracts, 4714 articles were rejected. From that search, full texts of 41 papers were assessed for eligibility, after which 22 papers were selected for the review. Five additional articles were obtained based on the manual search of the reference lists that were also included in the review. Finally, 27 papers were found to be eligible for inclusion in this systematic review and meta-analysis.

Papers were classified according to the characteristics of the type of design and the sources of information in the following five subgroups of studies (Table 1, Table 2, Table 3, Table 4 and Table 5):

1. Self-reported questionnaires in population samples (six articles) [10,11,12,13,14,15]
2. Cohort studies (four articles) [16,17,18,19]
3. Population-based integrated information systems (seven articles): including ambulatory and hospital discharge data that permit to calculate
  - Incidence (six articles) [20,21,22,23,24,25]
  - Prevalence (five articles) [21,22,23,24,26]
4. Hospital discharge databases (six articles) [27,28,29,30,31,32]
5. Other designs (four articles) [33,34,35,36]

According to the Joanna Briggs Institute Critical Appraisal Checklist, among 14 studies with prevalence data, 3 had a high risk of bias, 3 had a moderate risk of bias, and 8 were studies with a low risk of bias (Table 6). Most of the prevalence studies with a moderate or high risk of bias had a lack of explaining reasons for nonresponse rates and used self-reported endometriosis, potentially increasing measurement bias (under/overreporting); they also did not describe possible confounding factors in the analysis.

The main characteristics of the studies included in this review are shown in Table 1. The papers were published between 1989 and 2019 and originated from Norway [10], Denmark [11], the USA [12,16,19,20,25,26,33,35], Australia [13,14], Puerto Rico [15], Sweden [17], Japan [18], the UK [21,22], Israel [23], Germany [24], Iceland [27], Finland [28], Canada [29], Italy [30,32,34,36], and France [31]. The age of participating women varied in different studies from the minimal age of 10 years and older. The diagnosis of endometriosis was established mostly based on the diagnostic codes of International Classification of Diseases (ICD) [17,20,21,22,23,24,25,26,27,28,29,30,31,32], self-reported data [10,11,12,13,14,15,16,18,19,35], and by surgery or image tests [33,36] or a combination of self-reported data and image tests [34].

Publications based on self-reported data included women of reproductive age. Those studies included typically large samples, but response rates varied. Some of those studies actually focused on endometriosis or other gynecological issues, whereas others addressed broad health issues, and endometriosis was only part of their interest.

Incidence data were obtained from cohort studies that did not focus on endometriosis or gynecological problems but general health. The Nurses’ Health Study II is among the largest investigations into the risk factors for major chronic diseases in women that includes regular follow up of study participants since 1976 and repeated assessment of health and lifestyle factors. The Japan Nurses’ Health Study is a prospective cohort to study the effects of lifestyle and healthcare on women’s health, estimate the effects of those factors on their health, and establish evidence regarding the risks and benefits of treatments. The study was initiated in 2001. These three studies required clinical confirmation of endometriosis after being first self-reported by participating women. The Uppsala Birth Cohort Multigeneration Study, established in 2004, combines existing data on a representative and well-defined cohort of 14,192 males and females born in Uppsala from 1915 to 1929, with information on descendants using routine data registers.

The next category of studies comprises those that extract data on endometriosis diagnosis and treatments from integrated population-based datasets. The Rochester Epidemiology Project is a collaboration of clinics, hospitals, and other medical facilities that have agreed to share their medical records and allow researchers to study health and illnesses in the people living in this community. The United Kingdom General Practice Research Database is a computerized database of anonymized data from patient records from approximately 3.6 million patients, equivalent to about 6% of the population, collected from over 480 general practices. The Health Improvement Network contains anonymized longitudinal patient records for approximately 6% of the population of the United Kingdom.

The Defense Medical Surveillance System is a continuously expanding relational database that documents medical experiences of members from US Armed Forces members throughout their careers. It contains up-to-date and historical data on diseases and healthcare events (e.g., hospitalizations, ambulatory visits, laboratory diagnosis, treatments). Three other authors analyzed data extracted from specific health insurance databases. The definition of endometriosis included several criteria combining data both from ambulatory and hospital care.

Studies reporting data from hospital discharges exclusively defined endometriosis included in occasions the need for confirmation exploring data existing in medical records as histological confirmation.

In the last group, there are a series of studies whose designs and sources of data did not fit any of the types listed above. Interestingly, and regardless of the differences in methodology and sample size, the three that estimated prevalence provided roughly similar estimates: 3.2–4.7%.

Table 1, Table 2, Table 3, Table 4 and Table 5. Characteristics of the included studies for systematic review and meta-analysis of prevalence and incidence of endometriosis.

### 3.1. Pooled Prevalence and Incidence Rate of Endometriosis

The prevalence of endometriosis varied between 0.01 and 0.08, whereas the annual incidence rate of endometriosis was mostly between the range of 0.45 and 3.90 per 1000 PE, with the highest rate of 6.80 per 1000 PE in the study of Yasui and colleagues [18].

**Figure 2:**
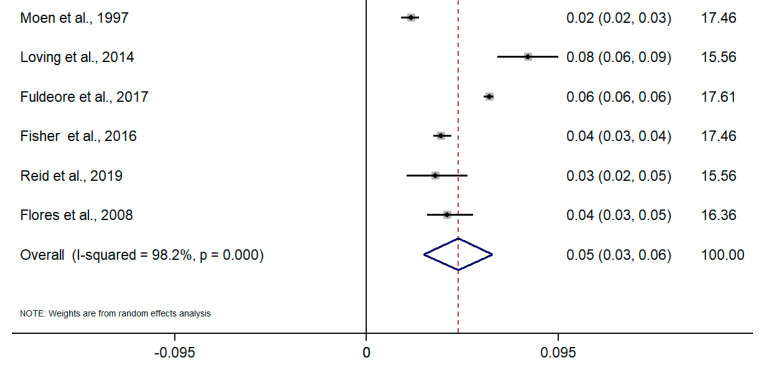
Meta-analysis of the prevalence of endometriosis based on self-Reported questionnaires. Note: black lines with squared dots—the prevalence with confidence intervals for each individual study; rhombus—pooled prevalence with confidence interval; red dotted line—line of no effect (studies crossing the line are not statistically different).

**Figure 3:**
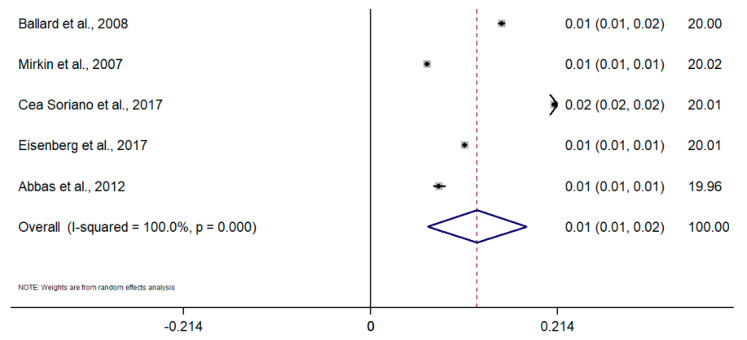
Meta-analysis of the prevalence of endometriosis based on population-based integrated information systems. Note: black lines with squared dots—the prevalence with confidence intervals for each individual study; rhombus—pooled prevalence with confidence interval; red dotted line—line of no effect (studies crossing the line are not statistically different).

**Figure 4:**
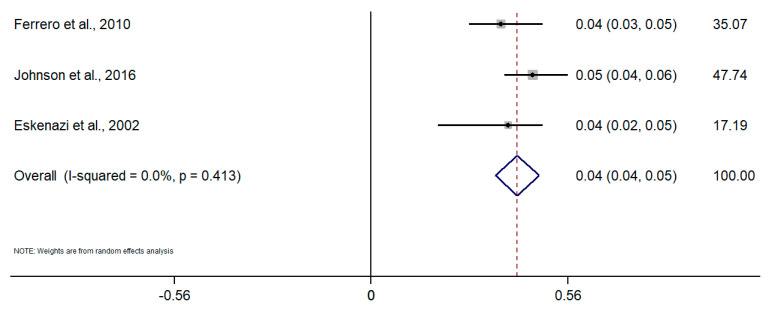
Meta-analysis of the prevalence of endometriosis based on studies using data from other sources. Note: black lines with squared dots—the prevalence with confidence intervals for each individual study; rhombus—pooled prevalence with confidence interval; red dotted line—line of no effect (studies crossing the line are not statistically different).

**Figure 5:**
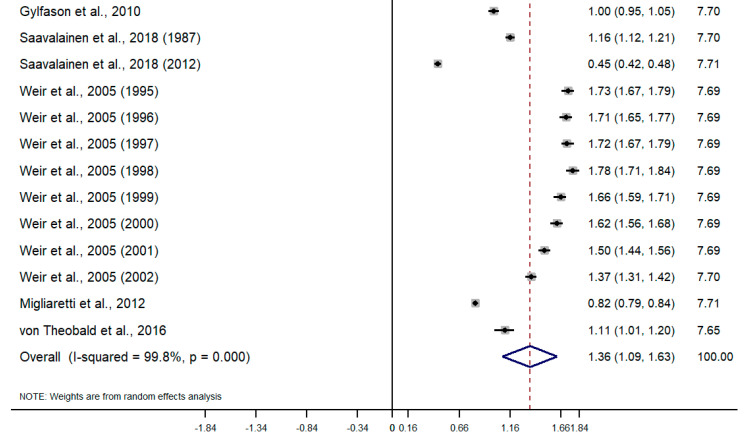
Meta-analysis of the incidence of endometriosis per 1000 person-years based on hospital discharge databases. Note: black lines with squared dots—the prevalence with confidence intervals for each individual study; rhombus—pooled prevalence with confidence interval; red dotted line—line of no effect (studies crossing the line are not statistically different).

**Figure 6:**
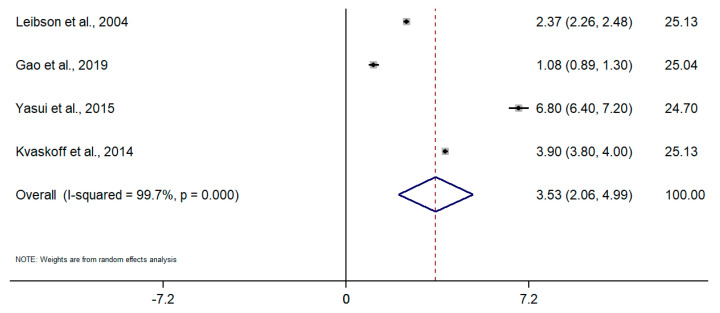
Meta-analysis of the incidence of endometriosis per 1000 person-years based on the data from cohort studies. Note: black lines with squared dots—the prevalence with confidence intervals for each individual study; rhombus—pooled prevalence with confidence interval; red dotted line—line of no effect (studies crossing the line are not statistically different).

**Figure 7:**
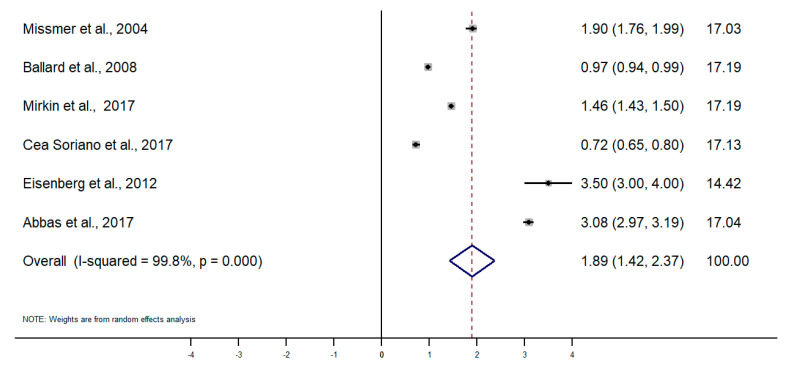
Meta-analysis of the incidence of endometriosis per 1000 person-years based on population-based integrated information system data. Note: black lines with squared dots—the prevalence with confidence intervals for each individual study; rhombus—pooled prevalence with confidence interval; red dotted line—line of no effect (studies crossing the line are not statistically different).

Taking into account the difference in the type of design and sources of information among studies, random-effects meta-analyses were conducted separately for each subgroup. To calculate the meta-analysis of the incidence and prevalence of endometriosis, the prevalence and incidence, as well as their standard errors (or calculated from confidence intervals where applicable), were retrieved from selected articles. The results of the five meta-analyses are the following (Figure 2, Figure 3, Figure 4, Figure 5, Figure 6 and Figure 7). The meta-analysis for studies based on self-reported data demonstrated a pooled prevalence of 0.05 (95% CI: 0.03; 0.06) (Figure 2); for studies with data from population-based integrated information systems, it was 0.01 (95% CI: 0.01; 0.02) (Figure 3); and 0.04 (95% CI 0.04; 0.05) in studies using other sources of data (Figure 4).

The pooled incidence rate of endometriosis for studies based on hospital discharges was 1.36 per 1000 PY (95% CI: 1.09; 1.63) (Figure 5); for cohort studies, it was 3.53 per 1000 PY (95% CI: 2.06; 4.99) (Figure 6); and for population-based integrated information systems, it was 1.89 per 1000 PY (95% CI: 1.42; 2.37) (Figure 7). Additionally, the meta-analysis indicated the high heterogeneity based on I-squared statistics between 98.2 and 100.0% (*p* < 0.001) for subgroups of studies.

### 3.2. Publication Bias

**Figure A1:**
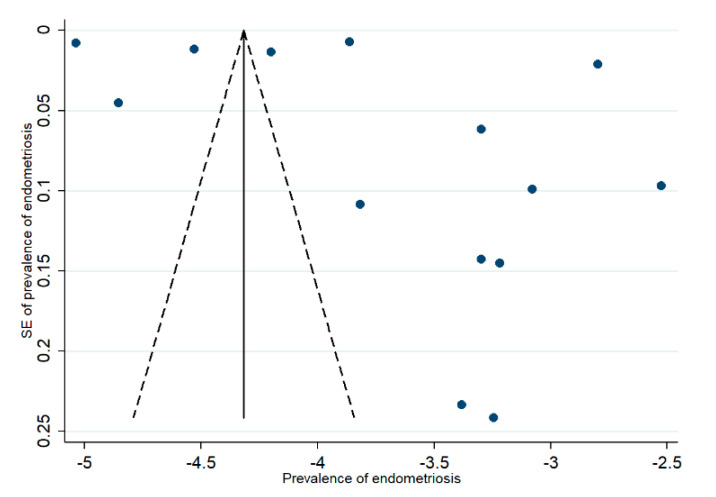
Funnel plot for prevalence data. Note: blue dots represent individual studies; the outer dotted line indicates the triangular region within which 95% of studies are expected to lie in the absence of both biases and heterogeneity; the solid vertical line represents the line of no effect, derived using fixed-effect meta-analysis.

**Figure A2:**
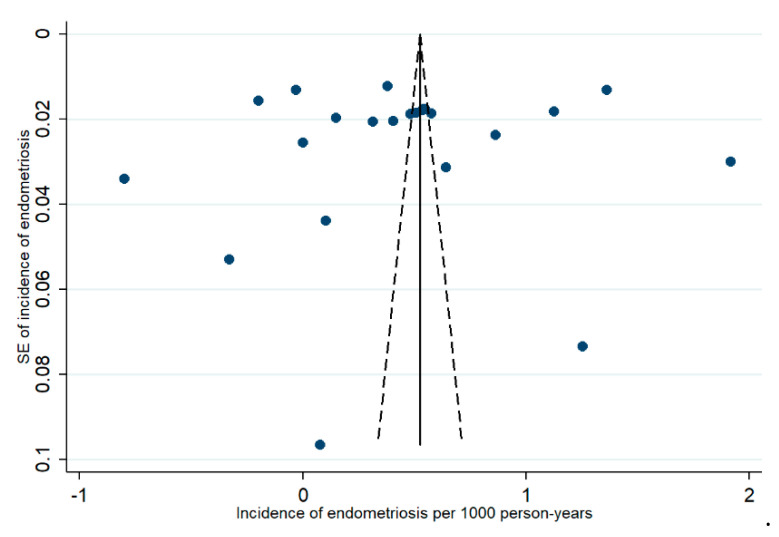
Funnel plot for incidence data. Note: blue dots represent individual studies; the outer dotted line indicates the triangular region within which 95% of studies are expected to lie in the absence of both biases and heterogeneity; the solid vertical line represents the line of no effect, derived using fixed-effect meta-analysis.

Publication bias of selected studies was assessed by funnel plots separately for prevalence and incidence data, which showed broad asymmetric results (Figure A1 and Figure A2). Egger’s test results indicated that this asymmetry was not statistically significant (*p* < 0.48 and *p* < 0.42, respectively).

## 4. Discussion

This work is the first review of the literature to systematically collect the evidence, obtain pooled estimates on the incidence and prevalence of endometriosis in general populations, and assess the strengths and limitations of the different data, methods, and case definitions. The main finding of this work is that the prevalence of endometriosis appears to have a range between 1 and 5% and an incidence between 1.4 and 3.5 per thousand person-years. These figures are below the classical statement that endometriosis has a prevalence of 10% in the population [6]. Nevertheless, this is a relevant second finding of this review, and differences in estimates were also found depending on the specific design, source of information, and case definition of the primary studies, with self-reported data having the highest prevalence estimations of over 5%; contrarily, studies using population based-clinical data reported prevalence rates below 1%. Incidence was lower in studies obtaining cases of patients discharged from hospitals, whereas higher incidence rates were obtained in cohort studies. Data were obtained from different countries that may use different recommendations and guidelines for the diagnosis of endometriosis, which may influence the prevalence and incidence estimates in those regions. These studies included women from different age groups, which also may contribute to the identified variations in the estimates. There were two studies utilizing data from hospital discharge databases and one study with self-reported endometriosis, which included women over 60 years (*n* = 425) and between 55 and 70 years of age (*n* = 747) and women older than 56 years (*n* = 471), respectively [11,28,29]. The main symptoms were infertility, peritoneal adhesions, genital pain, and ovarian cyst or growth.

One study showed an 11% incidence in a group of women age-matched from a group of women who had been indicated with residence in a 50-miles radius of participating clinical centers. However, this study did not report the size and characteristics of the population in which endometriosis was diagnosed or how those women were identified from that sample. Both did not properly explain why only about 50% of women who were screened finally received an MRI to diagnose endometriosis.

This review also conducted an appraisal of the quality of the works included, but the checklist could only be used in a limited number of papers, probably reflecting the specificities of the different designs included in this review. It is relevant to mention that the risk of bias measured by this tool indicates that the paper with the highest prevalence (8%) and those with the lowest (1%) show the best quality. However, the study reporting the highest prevalence while having a low bias score had a 48% response rate, which poses serious doubts regarding the validity of its estimated prevalence. A limitation of this review is that only one database was searched. No significant asymmetry was found reflecting no significant publication bias.

The disparate results obtained in the studies here included may not only be due to methodological issues and the specific limitations of the different designs and data analyzed, including case definitions and subject selection strategies, but also to the inherent heterogeneity and complexity of endometriosis.

The true incidence or prevalence of endometriosis is difficult to establish. Endometriosis is a heterogeneous clinical condition, with significant variability in terms of presentation and progression and an absence of specific biomarkers for its diagnosis and follow up, for which imaging tests do not allow identifying all cases with sufficient sensitivity and specificity [37,38]. Since visualization by endoscopy or laparotomy and histological confirmation is required for a definitive diagnosis, as a gold standard method, compared with a less accurate diagnosis by patient history, physical examination and noninvasive tests [7,39,40] make it difficult to arrive at a definite figure for its prevalence and incidence.

The problems for the identification of the prevalence or incidence of endometriosis were already described in the Eskenazi article, with very clear recommendations on how to carry out these studies [4]. However, more than 20 years later, those recommendations to understand the epidemiology of endometriosis have not yet been put into practice, so there remain significant methodological limitations to enable a definite characterization of the population magnitude of endometriosis.

Thus, it is relevant to note how when assessing the discrepancy in the estimate identified, these are usually higher in studies with surveys and self-reported data by women [7], whereas they are lower in cohort studies in which clinical confirmation was considered or those using population-based datasets that estimate endometriosis rates based on diagnostic codes of health care utilization databases, including both ambulatory diagnosed cases and hospital discharges.

Some of the studies with self-reported data had low response rates, a serious limitation to extract valid conclusions from those reports. It would be critical to know if there were differences in the characteristics of women who responded or not to those questionnaires, but this information is not reported in those papers. Self-reported data may be subject to certain bias; it has been suggested it may have a fairly good predictive ability to have an endometriosis diagnosis [41].

Studies that obtain prevalence or incidence estimates through automated clinical registries and calculate rates using ICD-type diagnostic codes may be subjected to the underreporting of certain diagnoses. However, it is important to note that these studies were carried out in countries and health systems with very diverse ethnic, cultural, organizational, or healthcare settings and still consistently report low incidence or prevalence figures that range between 0.1 and 0.2%. Publications reporting data exclusively from surgical cases may offer a limited view of the broad spectrum of the disease of those women who have a surgical intervention to remove endometrial tissue. Surgery is the only treatment that can completely remove the lesions associated with endometriosis. It is performed in the event of incapacitating symptoms and/or infertility. Studies based on integrated information systems include data extracted both from ambulatory care medical records, either from primary or specialized consultations, may be including cases with a clinical diagnosis and treatment for endometriosis regardless of histological or surgical confirmation.

A concern in the clinical management of endometriosis is the existence of delays in its diagnosis [42,43]. Although delays may affect women’s well-being, delays would only influence incidence or prevalence if they vary across settings, countries, or healthcare systems. If they are systematic, no effect should be assumed on incidence or prevalence. There is no evidence of the existence of variation in diagnostic delays, and the effect that this delay may have on the epidemiological burden of endometriosis is not clear [42,44], as no meta-analysis or systematic reviews investigating this question were conducted. However, single studies originating from Europe, Australia, China, and the US report diagnostic delays between 3.5 and 13 years [45,46,47,48,49], with the underrepresentation of years of diagnostic delays from Asian and African countries.

Living and working environments, along with regional and epigenetic factors, might also impact the development and manifestations of endometriosis, which were not taken into account in many prevalence and incidence studies. Their influence needs further investigation [16,50,51,52,53,54,55].

The presence of asymptomatic endometriosis could also not be ruled out. In this case, it is generally discovered incidentally when the patient seeks medical advice due to difficulty conceiving, as a large proportion of endometriosis patients are, in fact, infertile [56,57,58]. However, if women do not experience fertility problems asymptomatic, painless endometriosis may remain undetected. Although there may be differences between clinically diagnosed endometriosis and endometriosis diagnosed from surgery and/or histology, if endometriosis is asymptomatic, it would only be possible to be diagnosed in population studies not only through interviews or questionnaires, but it would ideally require at least a gynecological examination. The closest to this approach are cohort studies, such as the Nurses’ Study from the USA or the Uppsala study, in which annual incidence rates of approximately 0.2% are reported [16,17]. In the study by nurses from the Japan Nurses’ Health Study [18], although the reported incidence was much higher when clinical confirmation was investigated, the figures were substantially reduced.

Given the significant variability in the diagnostic and clinical management of endometriosis, it is not surprising the significant heterogeneity identified in the meta-analysis, even considering that separate random-effects models were conducted for the different types of designs used in this review. The criteria for the diagnosis of endometriosis could vary in studies from country to country, which could explain some variations in estimates. For example, ultrasound examination and a gold standard of diagnosis laparoscopy have different positive predictive values, subsequently leading to measurement bias in the studies and increased between-study variability [37]. Apart from this, there could be other methodological heterogeneity, including sampling strategy, recruitment methods, differences in demographic characteristics of study populations (age, ethnicity, and others). Results under the assumption of a random-effects model tend to be more conservative than those obtained assuming fixed effects, resulting in broader confidence intervals.

In this case, and even conducting separate subgroup analyses for specific types of papers, significantly elevated values of Q and I^2^ were obtained. One of the main criteria for deciding to conduct a meta-analysis is to check for heterogeneity, but in this case, instead of a limitation to extract conclusions regarding the pooled estimates of incidence and prevalence in this meta-analysis, heterogeneity may be a reflection that endometriosis is still a highly unknown and complex syndrome, with diverse clinical manifestations and progression [36].

## 5. Conclusions

Endometriosis is a heterogeneous clinical problem with significant uncertainty regarding its etiopathogenesis, diagnosis, treatment, and prognosis. It is well established that its main clinical manifestation, pain, causes a significant impact on women’s quality of life and represents a significant medical and social burden because of its direct and indirect costs. This work offers a comprehensive vision of the advantages and limitations of the various methodological approaches to provide estimates of the incidence or prevalence of endometriosis. The data provided by those works indicate a pooled estimated prevalence of endometriosis at around 1–5% and an incidence between 1.4 and 3.5 per thousand per year. The heterogeneity in the designs and data analyzed, as well as the clinical complexity and difficulties for the diagnosis of endometriosis, may influence the variability in those estimates. As well as a necessity for improving the biomedical and clinical evidence bases for the diagnosis and clinical management of this condition, appropriately designed epidemiological studies remain necessary to provide a valid estimation of the population burden of endometriosis.

## Figures and Tables

**Table 1:** Questionnaires and self-reported data.

Article	Years of Follow up	Setting	Population of Reference	Age	Source of Information	Method of Diagnosis	Prevalence	Incidence
Moen and Schei, 1997 [10]	1992–1993	Sor-Trondelag, Norway	5139 invited, 4089 (79.6%) participating, 4034 (78.5%) returned the questionnaire	42–44	Questionnaire sent to women as part of a cardiovascular risk prevention program	Self-reported	2.2%	0.30%
Loving et al., 2014 [11]	2010–2011	Denmark	2500 (response rate of 48%)	≥18	Questionnaire sent by mail as part of a study on chronic pelvic pain	Self-reported	8%	
Fuldeore and Soliman, 2017 [12]	2012	USA	59,411 contacted 48,020 responded (response rate of 15.5%)	18–49	Questionnaire sent by email to 3 panels	Self-reported	6.1%	
Fisher et al., 2016 [13]	2012	Australia	7427	34–39	Australian Longitudinal Study of Women’s Health	Self-reported	3.7%	
Reid et al., 2019 [14]	2017	Australia	652	18–49	Questionnaire sent to a panel	Self-reported in the last 3 year	3.4%	
Flores et al., 2008 [15]		Puerto Rico	1285 (response rate of 93%)		Questionnaire in specific places	Self-reported	4%	

**Table 2:** Cohort studies.

Article	Years of Follow up	Setting	Population of Reference	Age	Source of Information	Method of Diagnosis	Incidence
Leibson et al., 2004 [16]	1989–1999	USA	726,205 women-year		Nurses’ Health Study II	Review of clinical reports of self-reported cases	237/100,000 women-year
Gao et al., 2019 [17]	1996–2008	Uppsala, Sweden	4429 identified 3476 with obstetric record 3406 included	Born 1933–1972	Uppsala Birth Cohort Multigenerational Study and a national registry of hospital discharges	Diagnostic codes	1.08 per 1000 women-year
Yasui et al., 2015 [18]	2001–2012	Japan	15,019		Japan Nurses’ Health Study	Questionnaire sent to women that have reported endometriosis: confirmed with image or surgery	6.8% 1025 reported, 862 responded to the questionnaire, 638 diagnoses confirmed Image: 38.3% Surgery: 24.4%
Kvaskoff et al., 2014 [19]	1989	USA	A cohort of 116,430 female US nurses (response rate >90%)	25–42	Nurses’ Health Study II	Self-reported laparoscopically confirmed endometriosis	3.9 per 1000 person-years

**Table 3:** Population-based integrated information systems.

Article	Years of Follow up	Setting	Population of Reference	Age	Source of Information	Method of Diagnosis	Prevalence	Incidence
Missmer et al., 2004 [20]	1987–1992	County Olmsted, USA		≥15	Rochester Epidemiology Project	Diagnostic codes and review of medical records		1.9 per 1000 women-year (85% surgical confirmation)
Ballard et al., 2008 [21]	1992–2001	UK		15–55	United Kingdom General Practice Research Database	Diagnostic codes from primary care and hospitals	1.5%	0.97/1000 women-year 0.77/1000 women-year with cases “definitive” and “probable”
Mirkin et al., 2007 [26]	1999–2003	USA	6,220,349	18–55	Private insurance database	Diagnostic codes	0.7%	
Cea Soriano et al., 2017 [22]	2000–2010	UK	4,990,621 person-years	12–54	The Health Improvement Network and the Hospital Episode	Diagnostic codes and review of medical records	2.1%	1.5 per 1000 women-year
Eisenberg et al., 2018 [23]	2000–2015	Israel	6146	15–55	Private insurance database	Diagnostic codes	10.8 per 1000	7.2 per 10,000
Abbas et al., 2012 [24]	2004–2008	Hesse, Germany	Prevalence: 62,323 Incidence: 54,364	15–54	Private insurance database	Diagnostic codes	7.8 per 1000	3.5 per 1000
Stahlman et al., 2017 [25]	2012–2016	USA	Women in active service Army, USAF, Navy y Marines		Electronic medical record of Defense Medical Surveillance System	Diagnostic codes		30.8 per 10,000 women-year

**Table 4:** Hospital discharges.

Article	Years of Follow up	Setting	Population of Reference	Age	Source of Information	Method of Diagnosis	Incidence
Gylfason et al., 2010 [27]	1981–2000	Iceland	1,303,815	15–49	Hospital discharge registry	Diagnostic codes and review of medical records	Surgical confirmation: 10.0 histological: 5.7 10,000 women-year
Saavalainen et al., 2018 [28]	1987–2012	Finland	Women included in the Finnish Population Register Center	10–84	National hospital discharge registry	Diagnostic codes	45–116 per 100,000
Weir et al., 2005 [29]	1994–2002	Ontario, Canada		15–70	National hospital discharge registry	Diagnostic codes	137–173 per 100,000
Migliaretti et al., 2012 [30]	2000–2005	Piamonte, Italia	3929	18–45	Regional hospital discharge registry	Diagnostic codes	81.8 per 100,000 women-year
von Théobald et al., 2016 [31]	2008–2012	France	14,239,197	15–49	National hospital discharge registry	Diagnostic codes	0.4–1.6%
Morassutto et al., 2016 [32]	2011–2013	Friuli Venezia Giulia, Italia	807,050 without a diagnosis in the previous 10 years	15–50	Regional hospital discharge registry	Diagnostic codes and histological confirmation	0.11% Histologically confirmed: 0.07% Prevalence: 1.14%

**Table 5:** Other designs.

Article	Years of Follow up	Setting	Population of Reference	Age	Source of Information	Method of Diagnosis	Prevalence	Incidence
Louis et al., 2011 [33]	2007–2009	California, Utah, USA	127	18–44	Population controls	Image		11%
Ferrero et al., 2009 [34]	2009	Local general practitioner clinic, Italy	1612 met inclusion criteria, 1291 were agreed to participate and complete questionnaire	Reproductive age <50 years	Questionnaire, medical records	Clinical exploration, surgery, image	3.7%	
Johnson et al., 2016 [35]	1999–2001	Flight attendants and school teachers from Detroit, Miami, and Seattle	1945 flight attendants and 236 school teachers	18–45	Reproductive health occupational assessment	Self-reported laparoscopic confirmed via telephone interview	4.67% 3.38%	
Eskenazi et al., 2002 [36]	20 years of follow up after the 1976 factory explosion in Italy	Seveso, Italy	601	Women who were ≤30 years old in 1976	Retrospective cohort study	Surgically confirmed disease or an ultrasound consistent with endometriosis	3.2%	

**Table 6:** Results from the Joanna Briggs Institute Critical Appraisal Checklist for studies reporting prevalence.

#	Author, Year	Q1	Q2	Q3	Q4	Q5	Q6	Q7	Q8	Q9	Q10	Total	Risk of Bias
1	Moen and Schei, 1997 [10]	No	Yes	Yes	Yes	No	No	No	Yes	No	NA	44.4%	High
2	Loving et al., 2014 [11]	Yes	Yes	Yes	Yes	Yes	No	Yes	Yes	No	NA	77.8%	Low
3	Fuldeore and Soliman, 2017 [12]	Yes	Yes	Yes	Yes	No	No	No	Yes	No	NA	55.6%	Moderate
4	Fisher et al., 2016 [13]	No	Yes	Yes	Yes	No	No	No	Yes	No	NA	44.4%	High
5	Reid et al., 2019 [14]	No	Yes	No	Yes	No	No	No	Yes	Yes	NA	44.4%	High
6	Flores et al., 2008 [15]	Yes	No	Yes	Yes	No	No	Yes	Yes	No	NA	55.6%	Moderate
7	Ballard et al., 2008 [21]	Yes	NA	Yes	Yes	NA	Yes	Unclear	Yes	Yes	NA	87.5%	Low
8	Cea Soriano et al., 2017 [22]	Yes	NA	Yes	No	NA	Yes	Yes	Yes	No	NA	71.4%	Low
9	Eisenberg et al., 2018 [23]	Yes	NA	Yes	No	NA	Yes	Yes	Yes	No	NA	71.4%	Low
10	Abbas et al., 2012 [24]	Yes	NA	Yes	Yes	NA	Yes	Yes	Yes	No	NA	85.7%	Low
11	Mirkin et al., 2007 [26]	Yes	NA	Yes	No	NA	Yes	Yes	Yes	No	NA	71.4%	Low
12	Ferrero et al., 2009 [34]	Yes	Yes	Yes	Yes	Yes	Yes	Yes	Yes	No	NA	88.9%	Low
13	Johnson et al., 2016 [35]	Yes	No	Yes	Yes	No	No	No	Yes	Yes	NA	55.6%	Moderate
14	Eskenazi et al., 2002 [36]	Yes	Yes	No	Yes	Yes	Yes	Yes	Yes	Yes	NA	88.9%	Low

Q1—Was the sample representative of the target population? Q2—Were study participants recruited in an appropriate way? Q3—Was the sample size adequate? Q4—Were the study subjects and the setting described in detail? Q5—Was the data analysis conducted with sufficient coverage of the identified sample? Q6—Were objective, standard criteria used for the measurement of the condition? Q7—Was the condition measured reliably? Q8—Was there an appropriate statistical analysis? Q9—Are all important confounding factors/subgroups/differences identified and accounted for? Q10—Were subpopulations identified using objective criteria? Total = ΣYes/All Items; N/A, do not contribute to the overall sum.

## Data Availability

Not applicable.
